# Synergistic Inhibition of Acetylcholinesterase by Alkaloids Derived from Stephaniae Tetrandrae Radix, Coptidis Rhizoma and Phellodendri Chinensis Cortex

**DOI:** 10.3390/molecules24244567

**Published:** 2019-12-13

**Authors:** Xiang-Peng Kong, Etta Y.L. Liu, Zhi-Cong Chen, Miranda Li Xu, Anna X.D. Yu, Qi-Yun Wu, Ying-Jie Xia, Ran Duan, Tina T.X. Dong, Karl W.K. Tsim

**Affiliations:** 1 Shenzhen Key Laboratory of Edible and Medicinal Bioresources, HKUST Shenzhen Research Institute, Hi-Tech Park, Shenzhen 518057, China; xpkong@ust.hk (X.-P.K.); zc.chen@siat.ac.cn (Z.-C.C.); lxuae@connect.ust.hk (M.L.X.); qwuah@connect.ust.hk (Q.-Y.W.); yxiaas@connect.ust.hk (Y.-J.X.); duanran@ust.hk (R.D.); 2 Institute of Pharmaceutical & Food Engineering, Shanxi University of Chinese Medicine, 121 Daxue Road, Yuci District, Jinzhong 030619, China; 3 Division of Life Science and Center for Chinese Medicine, The Hong Kong University of Science and Technology, Clear Water Bay, Hong Kong, China

**Keywords:** acetylcholinesterase, fangchinoline, coptisine, berberine, synergistic effect

## Abstract

Alkaloids having acetylcholinesterase (AChE) inhibitory activity are commonly found in traditional Chinese medicine (TCM); for example, berberine from *Coptis chinensis*, galantamine from *Lycoris radiata*, and huperzine A from *Huperzia serrata*. In practice of TCM, Stephaniae Tetrandrae Radix (STR) is often combined with Coptidis Rhizoma (CR) or Phellodendri Chinensis Cortex (PCC) as paired herbs during clinical application. Fangchinoline from STR and coptisine and/or berberine from CR and/or PCC are active alkaloids in inhibiting AChE. The traditional usage of paired herbs suggests the synergistic effect of fangchinoline–coptisine or fangchinoline–berberine pairing in AChE inhibition. HPLC was applied to identify the main components in herbal extracts of STR, CR, and PCC, and the AChE inhibition of their main components was determined by Ellman assay. The synergism of herb combination and active component combination was calculated by median-effect principle. Molecular docking was applied to investigate the underlying binding mechanisms of the active components with the AChE protein. It was found that fangchinoline showed AChE inhibitory potency; furthermore, fangchinoline–coptisine/berberine pairs (at ratios of 1:5, 1:2, 1:1, and 2:1) synergistically inhibited AChE; the combination index (CI) at different ratios was less than one when *Fa* = 0.5, suggesting synergistic inhibition of AChE. Furthermore, the molecular docking simulation supported this enzymatic inhibition. Therefore, fangchinoline–coptisine/berberine pairs, or their parental herbal mixtures, may potentially be developed as a possible therapeutic strategy for Alzheimer’s patients.

## 1. Introduction

Acetylcholinesterase (AChE) is an enzyme that hydrolyzes acetylcholine in the nervous system [1], and it is a target enzyme for drug development in neurodegenerative disease, including Alzheimer’s disease (AD) [2]. AChE inhibitors, e.g., tacrine, donepezil, and rivastigmine, are used to improve cholinergic transmission and to restrain cognitive impairment progress, and they are the common drugs for treatment of AD. Because of the complexity of AD, AChE inhibitors only offer limited symptom relief and produce side effects in patients [3,4]. There is no specific cure for AD; therefore, it is important to develop effective and low-toxicity drugs by multidisciplinary approaches. Traditional Chinese medicine (TCM) has been used in treating diseases for thousands of years in China, and has proven effectiveness in brain diseases [5,6,7]. According to TCM theory, different herbs can be used together as paired herbs, which increases the drug efficiency. For example, the pairing of Acori Tatarinowii Rhizoma and Polygalae Radix is commonly employed in treating insomnia and forgetfulness, and the pairing of Astragali Radix and Angelicae Sinensis Radix is used for treatment of blood deficiency. Supporting this idea of paired herbs, the combination of Astragali Radix and Angelicae Sinensis Radix shows much stronger pharmacological properties compared to usage of either herb alone [8,9,10,11]. 

Stephaniae Tetrandrae Radix (STR; the dried root of *Stephania tetrandra* S. Moore), Phellodendri Chinensis Cortex (PCC; the dried bark of *Phellodendron chinense* Schneid.), and Coptidis Rhizoma (CR; the dried rhizome of *Coptis chinensis* Franch.) are three commonly used herbal medicines in TCM prescriptions, and they have been used in treatment of dementia for years, such as the herbal mixtures of Fangji Dihuang Tang, Huanglian Jiedu Tang, and Zhibai Dihuang Tang. In clinical practice, STR-CR (Hanfangji San in <<*Waitai Miyao*>>, Volume 11) and STR-PCC (Jiawei Ermiao Tang in <<*Yizong Jinjian*>>, Volume 19) are two paired herbs for treating pain, edema, and diabetes with symptoms of water-dampness retention, or damp-heat of middle and downward flow [12]. Alkaloids are the main active components in these herbs [13,14]. Bisbenzylisoquinoline alkaloids are the major type of alkaloids in STR, in which fangchinoline is a highly abundant and effective ingredient with functions of anti-inflammation, immune regulation, and analgesia [15,16]. Meanwhile, berberine and palmatine are two important isoquinoline alkaloids in CR and PCC with anti-inflammatory activity [13,17], and our previous research showed that their combination had a strong synergistic effect on AChE inhibition [18]. However, the AChE inhibitory activities of these herbs and their combinations have not been fully reported. Here, we identified alkaloids, i.e., fangchinoline, coptisine, and berberine in STR, CR, and PCC, which showed AChE inhibitory activity. Being commonly used paired herbs in TCM, we speculated that the representative alkaloids in STR–CR and STR–PCC might show synergistic inhibitory effects on AChE. Molecular docking simulation was conducted to investigate the binding sites of these alkaloids for synergistic inhibition.

## 2. Results

### 2.1. Determination of Alkaloids in Herbal Extracts

**Figure 1:**
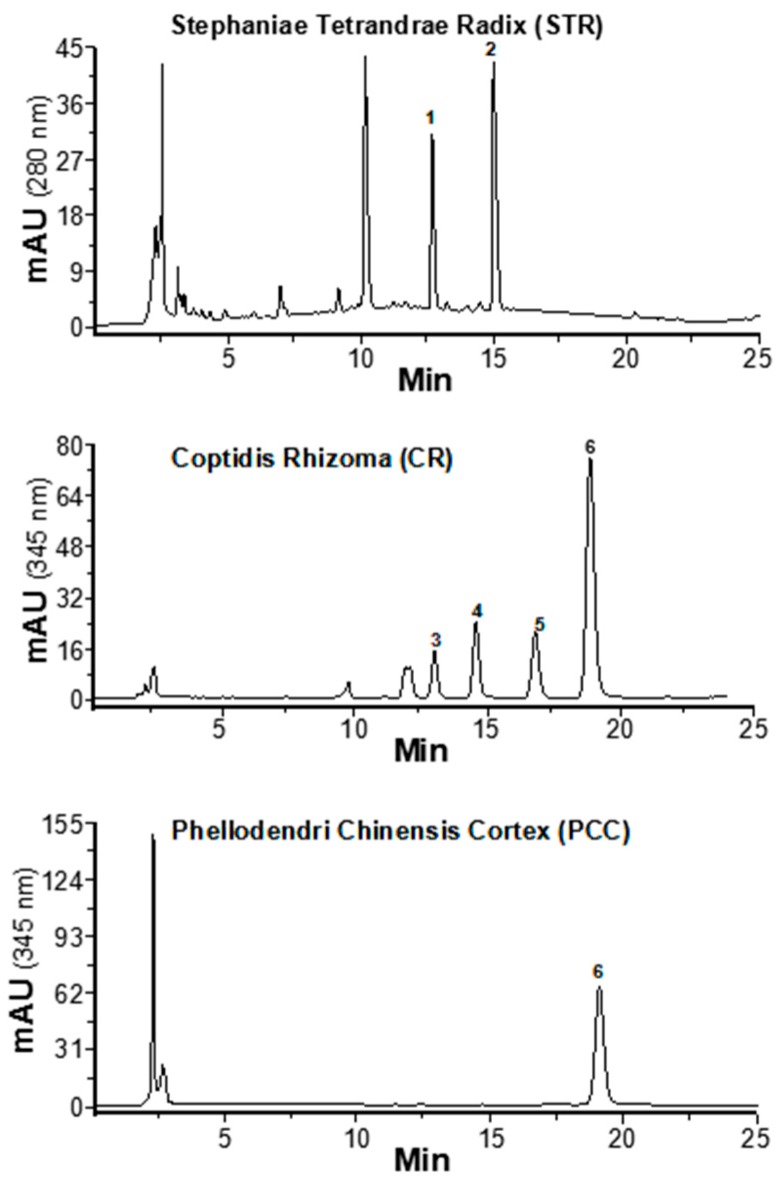
HPLC chromatograms of herb extracts. HPLC chromatograms of Stephaniae Tetrandrae Radix (STR), Coptidis Rhizoma (CR), and Phellodendri Chinensis Cortex (PCC) extracts: 1. Fangchinoline; 2. Tetrandrine; 3. Epiberberine; 4. Coptisine; 5. Palmatine; 6. Berberine. Typical profiles are shown here.

**Figure 2:**
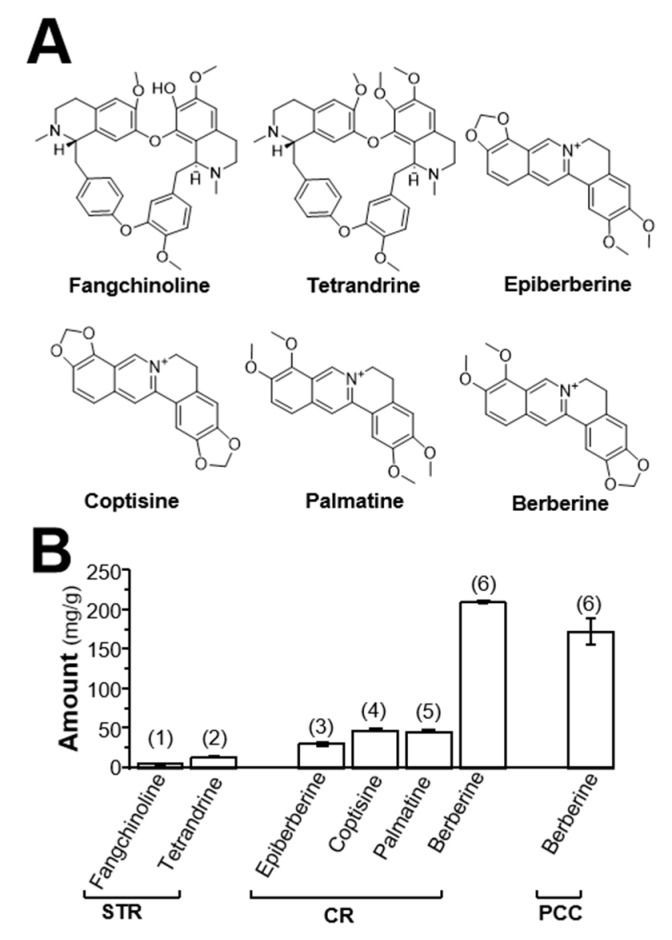
Structure and determination of alkaloids in STR, CR, and PCC. (**A**): Structure of alkaloids in STR, CR, and PCC extracts. (**B**): Determination of alkaloids in STR, CR, and PCC extracts. The numbers referring to different alkaloids are listed in Figure 1. Values are Mean ± SEM, *n* = 3.

Alkaloids are the main active components in STR, CR, and PCC. The HPLC chromatogram of the herbal extract is shown in Figure 1. The major alkaloids were identified in the fingerprints, and their amounts were determined. The main alkaloids’ structures are shown in Figure 2A. The linear relationships of alkaloid calibrations in STR, CR, and PCC are shown in Table 1, with the *R*^2^ of each component being >0.999. Fangchinoline and tetrandrine are two major bisbenzylisoquinoline alkaloids in STR extract, as seen in Figure 2B. Meanwhile, coptisine, berberine, epiberberine, and palmatine are the main isoquinoline alkaloids in CR extract. Similarly, berberine is the main active component in PCC extract. In addition, coptisine and berberine are two major alkaloids in CR and PCC, having higher amounts than the alkaloid in STR, as seen in Figure 2B.

### 2.2. Herbal Extracts and Their Alkaloids Inhibit AChE

In order to screen the active components from STR, CR, and PCC for AChE inhibition, the herbal extracts and their main alkaloids were tested in the brain lysate by Ellman assay, while tacrine was selected as a positive group. As shown in Table 2, STR extract inhibited AChE with an IC_50_ of 570.11 ± 10.41 μg/mL, while CR and PCC extracts showed AChE inhibition at IC_50_ values of 4.11 ± 0.15 and 22.19 ± 3.12 μg/mL, respectively. Fangchinoline showed strong AChE inhibitory potency with an IC_50_ value of 2.17 ± 0.05 μM, while tetrandrine did not show AChE inhibitory potency, suggesting that fangchinoline is the main AChE inhibitory component in STR. Coptisine, berberine, epiberberine, and palmatine were determined to be the main isoquinoline alkaloids in CR, which inhibited AChE with IC_50_ values at 13.50 ± 1.48, 2.33 ± 0.16, 18.7 ± 0.83, and 6.52 ± 0.84 μM, respectively. In order to reveal the synergy of inhibiting AChE by alkaloids in STR and CR, AChE inhibition by fangchinoline from STR, together with coptisine and/or berberine from CR, or PCC, was determined.

### 2.3. Herbal Combination Synergistically Inhibits AChE

**Figure 3:**
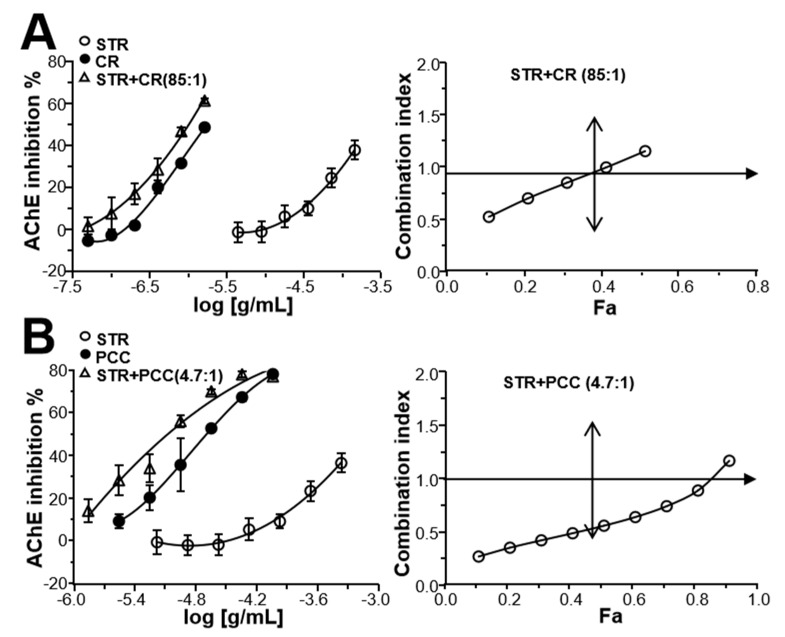
The synergistic effect of STR–CR/PCC pairs on AChE inhibition. (**A**): The synergistic effect of the STR–CR pair on AChE inhibitory activity. Left: STR, CR, and their combination dose-dependently inhibits AChE in vitro. Different doses of STR (6.6–418 μg/mL), CR (0.077–4.9 μg/mL), and their combination (85:1) were added to the assay solution and preincubated at 37 °C with the brain lysate, followed by the addition of ATCh (0.625 mM). Assay solution and the absorbance at 405 nm were measured. Right: Analysis of synergism of STR and CR evaluated by the median-effect principle. Data of AChE inhibitory activities were analyzed by the Chou–Talalay method as described in the Materials and Methods. The plots of Fa–CI in different assays were demonstrated. (**B**): The synergistic effect of the STR–PCC pair on AChE inhibitory activity. Left: STR (6.6–418 μg/mL), PCC (1.4–89.6 μg/mL), and their combination (4.7:1) dose-dependently inhibited AChE in vitro. Right: Analysis of synergism of STR and PCC evaluated by the median-effect principle. Data analysis was performed as for A, right. The data was calculated by inhibitory percentage of control, and expressed as Mean ± SEM, *n* = 3, each with triplicate samples.

To test the synergistic inhibition of STR–CR and STR–PCC, their combinations (concentration ratio for STR:CR was 85:1, and for STR:PCC was 4.7:1) were tested for AChE inhibition. These ratios were found to have excellent synergy (data not shown). The extracts of STR, CR, and PCC inhibited AChE in dose-dependent manners, with IC_50_ values of 570.11 ± 10.41, 4.11 ± 0.15, and 22.19 ± 3.12 μg/mL, respectively, as seen in Figure 3A,B, left. In addition, the STR–CR and STR–PCC combinations showed better inhibition in a dose-dependent manner, and the doses of fangchinoline and coptisine were markedly reduced at the same degree of AChE inhibition. The synergistic effects of STR-CR and STR-PCC combinations in AChE inhibition were further evaluated by the median-effect principle. As shown from the synergism analysis by the median-effect principle in Figure 3A right, when *F_a_* < 0.4, the CI value of the STR–CR combination is less than one, suggesting that its inhibitory effect is synergistic at low concentrations. Similarly, the STR–PCC combination showed better synergy in terms of AChE inhibition, as seen in Figure 3B right, CI < 1, when *F_a_* < 0.8.

### 2.4. Fangchinoline and Coptisine Synergistically Inhibit AChE

**Figure 4:**
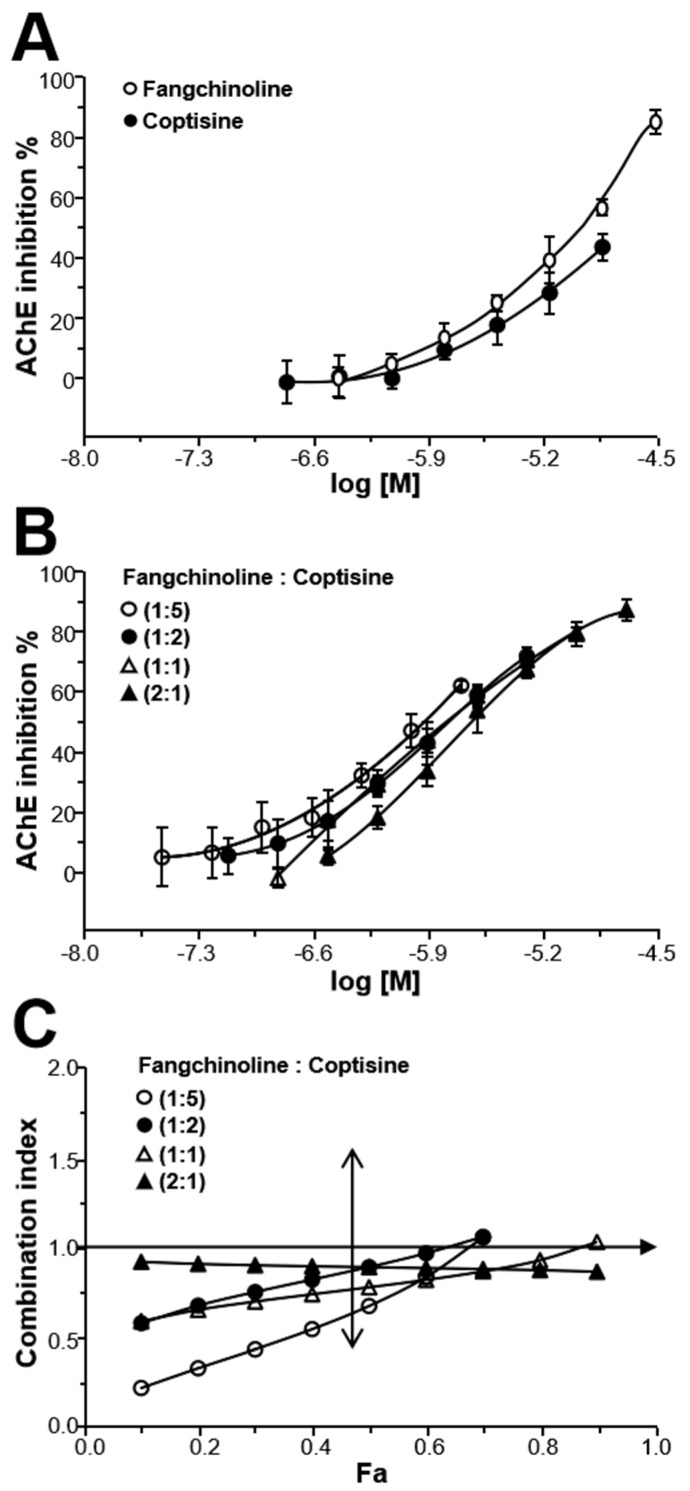
The synergistic effect of fangchinoline–coptisine pairs on AChE inhibition. (**A**): Fangchinoline (0.312–20 μM) and coptisine (0.156–10 μM) dose-dependently inhibited AChE in vitro. (**B**): Different ratios of fangchinoline–coptisine pairs (concentration ratio: 1:5, 1:2, 1:1, 2:1) dose-dependently inhibited AChE in vitro. The data was calculated by inhibitory percentage of control, and was expressed as Mean ± SEM, *n* = 3, each with triplicate samples. (**C**): Synergism analysis of fangchinoline–coptisine pairs (concentration ratio: 1:5, 1:2, 1:1, 2:1) was evaluated by median-effect principle. The AChE assay of fangchinoline, coptisine, and their combinations was performed as in Figure 3A, left. The synergism analysis of fangchinoline–coptisine pairs (concentration ratio: 1:5, 1:2, 1:1, 2:1) was performed as in Figure 3A, right.

Fangchinoline is a strong AChE inhibitor, and it is an abundant alkaloid in STR. Similarly, coptisine is a strong AChE inhibitor and an abundant alkaloid in CR, as seen in Figure 4A. To test synergistic inhibition by fangchinoline and coptisine, different ratios of their combinations (concentration ratio: 1:5, 1:2, 1:1, 2:1) were tested for AChE inhibition. As shown in Figure 4B, different ratios of fangchinoline and coptisine combinations showed good AChE inhibition in a dose-dependent manner. In addition, the AChE inhibitory potency of fangchinoline and coptisine combinations (1:5, 1:2, and 1:1) increased, with IC_50_ values (expressed as concentration of fangchinoline) of 1.18 ± 0.11, 1.71 ± 0.40, and 1.79 ± 0.44 μM at different ratios, respectively, which are much lower than when using either alkaloid alone.

The synergistic effect of fangchinoline and coptisine combinations on AChE inhibition was further evaluated by the median-effect principle. As shown from the Fa–CI plots in Figure 4C, when *F_a_* = 0.5, the CI values of fangchinoline and coptisine combinations (at ratios of 1:5, 1:2, 1:1, and 2:1) were 0.66, 0.88, 0.76, and 0.88, respectively, suggesting synergy between fangchinoline and coptisine at different concentration ratios. Except at the ratio of 2:1, the combinations of fangchinoline and coptisine showed better AChE inhibitory potency at low concentration when *F_a_* < 0.7. However, when *F_a_* > 0.8, the CI values of their combinations were close to or greater than one, indicating that the inhibitory effect of their combinations might be addictive or antagonistic.

### 2.5. Fangchinoline and Berberine Synergistically Inhibit AChE

**Figure 5:**
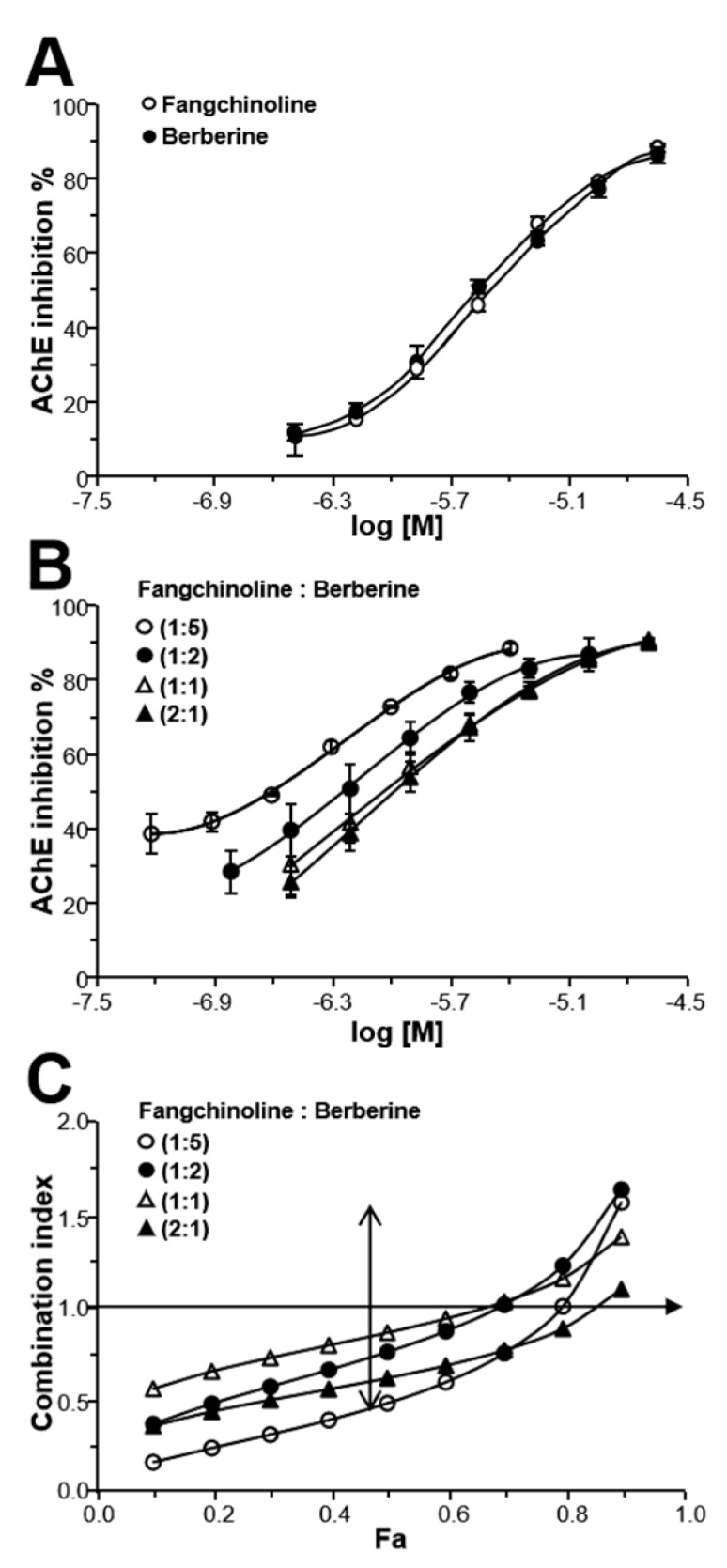
The synergistic effect of fangchinoline–berberine pairs on AChE inhibition. (**A**): Fangchinoline (0.312–20 μM) and berberine (0.312–20 μM) dose-dependently inhibited AChE in vitro. (**B**): Different ratios of fangchinoline–berberine pairs (concentration ratio: 1:5, 1:2, 1:1, 2:1) dose-dependently inhibited AChE in vitro. The data was calculated by inhibitory percentage of control, and was expressed as Mean ± SEM, *n* = 3, each with triplicate samples. (**C**): Synergism analysis of fangchinoline–berberine pairs (concentration ratio: 1:5, 1:2, 1:1, 2:1) was evaluated by the median-effect principle. The AChE assay of fangchinoline, berberine, and their combinations was performed as in Figure 3A, left. The synergism analysis of fangchinoline–berberine pairs (concentration ratio: 1:5, 1:2, 1:1, 2:1) was performed as in Figure 3A, right.

Berberine is a well-known AChE inhibitor existed in many herbal medicines, particularly in CR and PCC. Fangchinoline and berberine inhibited AChE in a dose-dependent manner, as seen in Figure 5A. To test the synergistic inhibition of fangchinoline and berberine, different ratios of their combinations (1:5, 1:2, 1:1, 2:1) were tested for AChE inhibition. The different ratios of fangchinoline and berberine combinations showed AChE inhibition in a dose-dependent manner, as seen in Figure 5B. In addition, the AChE inhibitory potency of fangchinoline and berberine combinations (1:5, 1:2, and 2:1) increased largely, with IC_50_ values (expressed as concentration of fangchinoline) of 0.19 ± 0.02, 0.55 ± 0.17, and 0.79 ± 0.47 μM, respectively, and the efficiency doses of fangchinoline and coptisine were markedly reduced, as compared to either compound alone. 

The synergistic effect of fangchinoline and berberine on AChE inhibition was further evaluated by the median-effect principle. When *F_a_* = 0.5, the CI values of fangchinoline and berberine combinations (at ratios of 1:5, 1:2, 1:1, and 2:1) were 0.46, 0.73, 0.84, and 0.59, respectively, as seen in Figure 5C. When *F_a_* < 0.7, the CI values of fangchinoline and berberine combination were less than one at ratios of 1:5, 1:2, and 2:1, which indicated a synergistic effect between fangchinoline and berberine at different concentration ratios; similarly, when *F_a_* < 0.5, the CI value of their combination was less than one at the ratio of 1:1, indicating synergistic action. However, when *F_a_* > 0.8, the CI values of fangchinoline–berberine were above one, indicating that the inhibitory effect of their combination might be antagonistic.

### 2.6. AChE Binding Sites Analysis of Fangchinoline, Coptisine and Berberine

**Figure 6:**
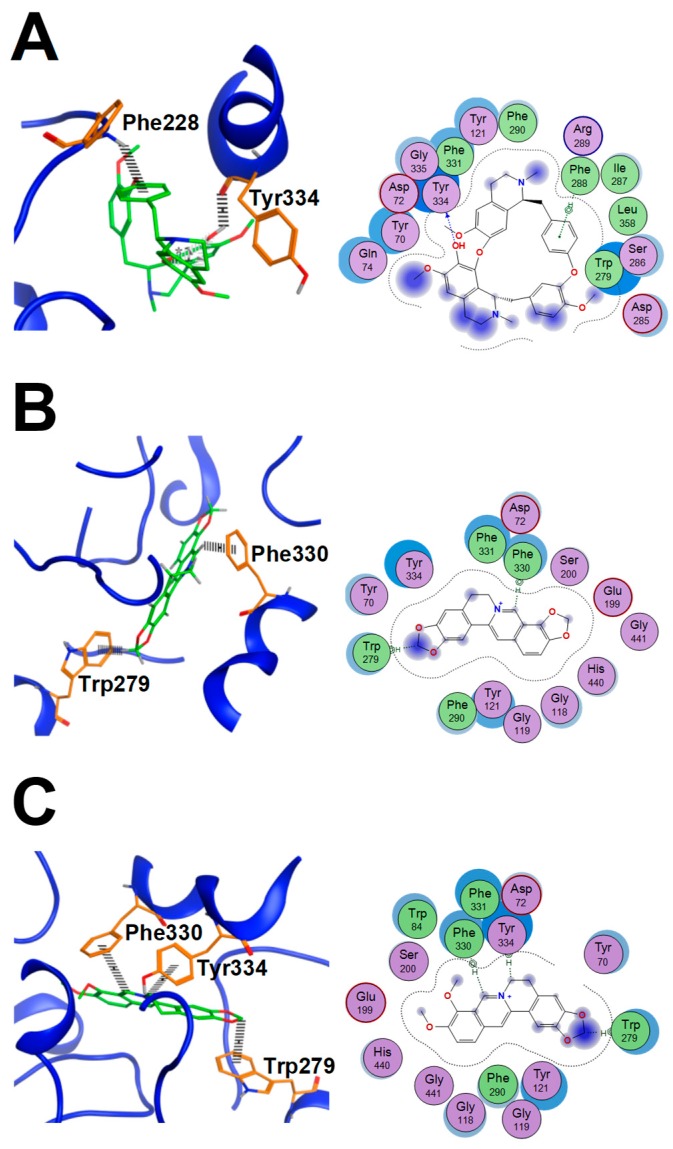
The binding modes of fangchinoline, coptisine, and berberine with AChE. (**A**). 3D interactions of fangchinoline with AChE (ligand is shown as green) and 2D interactions of fangchinoline with AChE. (**B**). 3D interactions of coptisine with AChE (ligand is shown as green), and 2D interactions of coptisine with AChE. (**C**). 3D interactions of berberine with AChE (ligand is shown as green) and 2D interactions of berberine with AChE.

According to the docking simulation results, the binding modes of fangchinoline, coptisine, berberine, and donepezil with AChE (PDB code: 1eve) were different in terms of their interactions with the AChE binding pockets. As shown in Table 3, the fitting scores of fangchinoline, coptisine, and berberine were −7.11 ± 0.32, −6.76 ± 0.02, and −7.00 ± 0.01, respectively, and it was consistent with their AChE inhibitory activities. As shown in Figure 6A, there is an H-donor interaction (2.59 Å) between hydroxyl oxygen atoms in the benzene ring of fangchinoline and TYR_334_ (peripheral anionic site, PAS active site) of the AChE protein, and a pi–H interaction (4.15 Å) between a pi bond of the aromatic ring in fangchinoline and PHE_2__88_ (the acyl pocket active site) of AChE. As for coptisine and berberine, as seen in Figure 6B,C, there are the same H–π interactions (3.99 and 4.03 Å) between hydrogen atoms in the nitrogen heterocyclic aromatic ring and PHE_330_ (gated flexible residues) of AChE. Similarly, there are other H–π interactions (3.90 and 3.67 Å) between hydrogen atoms in the methene group of five oxygen heterocyclic rings and TRP_279_ (the PAS active site) of AChE. However, there is an H–π interaction (3.57 Å) between hydrogen atoms in the methene group of berberine’s six-membered heterocyclic nitrogen ring and TYR_334_ (PAS active site) of AChE. Therefore, coptisine and berberine have a similar binding model with AChE’s active pocket, and berberine has more intermolecular interaction with AChE via active binding. Coptisine and berberine can combine with the gated flexible residue PHE_330_ of AChE, while fangchinoline has no such binding site. Donepezil is a commonly used therapeutic drug in AD treatment [19]. As shown in Table 3, donepezil can bind with the anionic subsite (TRP_84_) via H–π interaction (4.06 Å), PAS active site (TYR_334_ and TRP_279_) by H–π interactions (4.13, 3.45/4.03 Å), and the gated flexible active site (PHE_330_) by π–π interaction (3.54 Å). Interestingly, the binding of fangchinoline, coptisine, or berberine with AChE’s active site is largely consistent with that of donepezil. Hence, it is speculated that the combination of fangchinoline, coptisine, or berberine may have a synergistic effect on AChE inhibition.

## 3. Discussion

AD is an intractable neurodegenerative disease, and it is characterized by cognitive disorder and brain atrophy with histopathologic changes of senile plaques (SP), neurofibrillary tangles (NFT), cholinergic neurons loss, and gliosis with chronic inflammation [20,21,22,23]. The pathogenesis of AD is complex. The hypotheses of cholinergic defects, Aβ oligomerization, and τ-protein hyperphosphorylation have been proposed [21,24,25]. According to the cholinergic hypothesis, the cholinergic function of the hippocampus and basal ganglia in the forebrain is closely related to memory and cognition. Cholinergic deficiency, i.e., cholinergic nerve cell destruction, AChE activity elevation, and acetylcholine level reduction in synaptic cleft, has been identified in AD patients [26]. Hence, it is speculated that increased levels of neurotransmitters could enhance synaptic transmission, ameliorate impaired memory, and restrain the progress of cognitive impairment in AD patients [27]. AChE is an enzyme that hydrolyzes acetylcholine in the nervous system, which has two isoforms in the brain, i.e., a monomer (G1) and a tetramer (G4). The G4 isoform represents the majority of total AChE, and the change of the G4 isoform is closely related to cognition [28,29,30]. Interestingly, AChE can accelerate and induce Aβ oligomerization by forming stable complexes with molecular partners [31,32,33,34]. AChE plays an important role in the cholinergic anti-inflammatory pathway (CAP) by modulating the relationship of ACh and α7nAChR [35,36], which can participate in regulating immunity in the brain. Different kinds of AChE inhibitors have been developed for treatment of AD. However, they only offer limited symptom treatment, due to drug toxicity and the complexity of AD.

In clinical practice, the treatment of AD with TCM has shown good efficiency [37,38,39]. TCMs have been used in treating mental-related problems for thousands of years in China, and many of them show good effect in tranquilizing the mind, relieving anxiety, and enhancing learning and memory. According to the theory of TCM, mental state, emotional activities, especially high-level intellectual activities (e.g., planning and making decisions), are closely related to the functions of the liver and kidney. TCM herbal decoctions could have overall and comprehensive therapeutic effects by matching the corresponding herbs under the guidance of syndrome differentiation [38,39], e.g., the “liver-fire” excess symptom (showing forgetfulness, muttering, flushing, bitter taste, irritability) of AD patients could be relieved by therapies of clearing “liver-fire” and relieving depression when paired with CR and Gardeniae Fructus (as in Huanglian Jiedu Tang), and the sea of medulla insufficiency with “damp-heat” of downward flow symptoms (showing forgetfulness, lumbar debility, deep-colored urine, or difficult, painful urination) could be relieved by therapies of nourishing yin and reducing pathogenic fire of downward flow when paired with PCC and Rehmanniae Radix (Zhibai Dihuang Tang). In current clinical application, a herbal mixture of STR and Rehmanniae Radix (Fangji Dihuang Tang) is used for treatment of vascular dementia by therapies of nourishing yin and promoting diuresis. Among these therapies, herbal mixtures of CR–Gardeniae Fructus, PCC–Rehmanniae Radix, and STR–Rehmanniae Radix are effective paired herbs in AD treatment [17,37]. Here, we propose another herb pair: STR + CR/PCC can be tailored for AD treatment, as the contained alkaloids show synergistic effects towards AChE inhibition. 

Alkaloids are a group of naturally occurring compounds in medicinal herbs. There are different varieties of alkaloids in TCM, e.g., galantamine from *Lycoris radiata*, berberine from *Coptis chinensis*, and huperzine A from *Huperzia serrata.* Many of the alkaloids in TCMs have AChE inhibitory activity and show anti-inflammatory activity [40,41,42]. Out of the 150 alkaloids that we tested, 60 showed AChE inhibition. Plant alkaloids are known to have toxicity; however, the alkaloids from TCM could provide more information on their pharmacodynamic effect and toxicity, as their originated herbal medicines have been used in clinics for many years. Here, STR, CR, and PCC are three commonly used TCM herbs in Asia/China. According to the theory of TCM, STR is commonly used in promoting diuresis and clearing damp heat, and CR and PCC are commonly used in clearing damp heat of middle and downward flow. They have been used in dementia treatment for many years at dosages of 6–24 (STR), 9–12 (CR) and 6–12 (PCC) grams [43,44,45]. Fangchinoline is a bisbenzylisoquinoline alkaloid in STR, while coptisine and berberine are isoquinoline alkaloids in CR and PCC. Here, our results supported the clinical usage of STR–CR/PCC as paired herbs. More important, fangchinoline–coptisine/berberine combinations (at ratio of 1:5) could produce similar levels of effect at much lower dosage than that of either alkaloid alone, which suggests that the usage of fangchinoline–coptisine/berberine (at ratio of 1:5) could be much better in terms of drug safety. Our results showed that the required dosage of STR was higher than CR and PCC in STR–CR/PCC paired herbs in terms of AChE inhibition, while the dosage of fangchinoline was lower than coptisine and berberine in fangchinoline–coptisine/berberine combinations (at ratio of 1:5). This could be closely related to the different contents of fangchinoline, coptisine, and berberine in the herbal extracts.

Computer-aided drug screening is a convenient and efficient method for lead compound discovery [46]. Here, molecular docking was used to study the AChE inhibition by alkaloids from STR, CR, and PCC. It is known that the active site of AChE [19] is a deep and narrow gorge with a catalytic site, a PAS active site, and an anionic subsite active site. AChE contains a gated flexible residue active site and acylation active sites. The gated flexible residue (PHE_330_) is responsible for substrate trafficking down the gorge, and the spatial orientations of PHE_330_ are different when complexed with different substrates or inhibitors. As seen from the docking results, both coptisine and berberine could bind with the gated flexible active site (PHE_330_) and PAS active site (TRP_279_), while fangchinoline could interact with the acyl active sites (PHE_288_) and PAS active site (TYR_334_). It is speculated that fangchinoline and coptisine/berberine could form a ternary complex with AChE by simultaneously binding the PAS active site and the acyl active site. In addition, the synergistic inhibition of AChE by using combinations of alkaloids is closely correlated with enhancement of the affinities between ligands and active sites. Meanwhile, donepezil could bind with the anionic subsite (TRP_84_), PAS active site (TYR_334_ and TRP_279_), and gated flexible active site (PHE_330_) by π–π interaction (3.54 Å). Hence, it could also be suggested that the fangchinoline–coptisine/berberine combination has a synergistic effect on AChE inhibition, since the binding of fangchinoline, coptisine, and berberine with AChE’s active site is similar to that of donepezil. 

AChE can also have noncholinergic effect, i.e., inducing Aβ oligomerization via the PAS active site (TRP_279_), or regulating inflammation by CAP. The main alkaloids in STR, CR, and PCC could alleviate Aβ-induced injury or have an antineuroinflammatory effect [12,47,48]. The alkaloids in STR, CR, and PCC can affect other factors related with AD: fangchinoline has a protective effect on cyanide-induced neurotoxicity [49,50]; coptisine ameliorates cognitive impairment by inhibiting indoleamine 2,3-dioxygenase [51]; and berberine shows multiple activities to relieve symptoms of AD [52]. In summary, the combination of fangchinoline–coptisine/berberine could provide a potential therapeutic strategy for AD treatment; however, it needs to be studied further.

## 4. Materials and Methods

### 4.1. Chemicals and Preparation of Herb Extracts for HPLC Determination

Fangchinoline (Lot S090409-10, purity 99%) and berberine (Lot S071016-5, purity 99%) were purchased from Chengdu Manster Biotechnology (Chengdu, China); coptisine (Lot DST170711-003, purity 99%) and epiberberine (Lot DST170711-109, purity 99%) were purchased from Chengdu Dester Technology (Chengdu, China); palmatine (Lot S0805239, purity 99%); tetrandrine (Lot S090601-5, purity 99%) was purchased from Chengdu Ruifensi Biotechnology (Chengdu, China). STR is the root of *S. tetrandra* from Jiangxi, China; CR is the rhizome of *C. chinensis* from Sichuan, China. PCC is the dried bark of *P. chinense* from Sichuan, China. The authentication of herbs was performed by Prof. Xiangping Pei from Shanxi University of Chinese Medicine. STR, CR, and PCC herbs were weighed appropriately. Each of them was decocted twice with water (1st, 1:10 w/v, 40 min. 2nd, 1:8 w/v, 30 min), and the filtrates were combined, concentrated to extract, and dried at low temperature.

### 4.2. Determination of Alkaloids in Herbal Extracts

The herb extracts of STR, CR, and PCC were weighed accurately and dissolved in 50% MeOH at concentration of 2.18, 0.40, and 0.40 mg/mL, respectively. The dissolved extract was filtered by a 0.22 μm Millipore filter, and the filtrate was subsequently collected for HPLC determination. The standards of fangchinoline, tetrandrine, coptisine, berberine, palmatine, and epiberberine were weighed accurately and dissolved to stock solution of 1 mg/mL by MeOH. Different volumes of fangchinoline and tetrandrine stocks were mixed to prepare the mixed stock solution of alkaloids in STR, with final concentrations of 100 and 200 μg/mL, respectively. Similarly, the mixed stock solution of alkaloids in CR and PCC was prepared in MeOH, containing epiberberine, coptisine, palmatine, and berberine at 20, 40, 75, 75, and 300 μg/mL, respectively. Then, the mixed stock solutions were diluted to series of working standards with MeOH for HPLC determination. Chromatographic analysis was performed by Waters 2695 HPLC equipped with a UV-VIS photodiode array detector. Sample separation was achieved on an Innoval C_18_ column (4.6 × 250 mm, 5 µm) with a constant flow rate of 1.0 mL/min at 25 °C. The mobile phase of STR was composed of MeCN (A) and water (B, 0.1% phosphoric acid), using a gradient elution of 10–30% at 0–20 min, 30–45% at 20–40 min. Detection was performed at 280 nm. The mobile phase of CR and PCC was composed of MeCN and 0.05 M KH_2_PO_4_ solution (containing 0.4 g sodium dodecyl sulfate/mL, the pH was adjusted to 4.0 by phosphoric acid). The analyses were detected at 345 nm. The injection volume was set at 10 µL.

### 4.3. Preparation of Herbal Extracts and Alkaloids for AChE Assay

STR, CR, and PCC herbal extracts were weighed accurately and dissolved to maximum concentration (83.6, 78.8, and 84.2 mg/mL, respectively) by DMSO. Fangchinoline, tetrandrine, epiberberine, coptisine, palmatine, and berberine were weighed accurately and dissolved to maximum concentration (50, 6, 25, 25, 50, and 25 mM, respectively) by DMSO. The combinations of STR–CR/PCC with concentration ratio of 85:1 and 4.7:1 were individually prepared by mixing appropriate volumes of their extracts stock solutions. The solutions of STR–CR/PCC combinations (with initial system concentrations 418:4.9 and 418:89.6 μg/mL, respectively) were diluted into six concentrations by DMSO, respectively. The combinations of fangchinoline–coptisine/berberine at concentration ratios of 1:5, 1:2, 1:1, and 2:1 were individually prepared by mixing appropriate volumes of fangchinoline and coptisine or berberine stock solutions. The solutions of fangchinoline–coptisine combinations (with initial system concentrations 4:20, 10:20, 20:20, and 20:10 μM, respectively) were diluted into seven concentrations by DMSO, respectively. The solutions of fangchinoline–berberine combinations (with initial system concentrations 2:10, 5:10, 10:10, and 20:10 μM, respectively) were diluted into seven concentrations by DMSO, respectively. The stocks of tetrandrine, epiberberine, and palmatine were taken appropriately and diluted into six concentrations (with initial system concentrations 30, 100, and 50 μM, respectively) by DMSO, respectively.

### 4.4. Ellman Assay

The mouse brain lysate (0.1 g/mL, containing 10 mM HEPES (pH 7.4), 1 mM EDTA, 1 mM EGTA, 150 mM NaCl, 0.5% triton X-100, 10 μg/mL leupeptin, 10 μg/mL aprotinin, 10 μg/mL pepstatin, and 50 μg/mL benzamidine HCL) was prepared. AChE enzymatic activity was assayed by 96-well microtiter plate with a final volume of 200 μL, according to the Ellman method [53,54]. The assay medium consisted of brain lysate (5 mg/mL) containing different concentrations of testing drugs, 80 mM Na_2_HPO_4_ (pH 7.4), 0.1 mM iso-OMPA, 0.625 mM ATCh, and 0.5 mM DTNB. Briefly, the mixture of brain lysate containing the testing drugs, Na_2_HPO_4_ buffer, and iso-OMPA solution were incubated at 37 °C for 10 min, and then ATCh and DNTB solutions were added. The reaction solution was incubated at 37 °C for 30 min, and AChE activity was determined by measuring the absorbance at 405 nm. To eliminate the drug–solvent influence on AChE enzymatic activity, the concentration of DMSO was controlled at 0.5% in the 200 μL reaction volume. In order to correct for color interference at 405 nm, a background color contrast group without brain lysate and a drug color contrast group without brain lysate were set, respectively. AChE activity percent of inhibition was calculated by the following equation: AChE activity inhibition (%) = (1 − absorbance with inhibitor/absorbance without inhibitor) × 100%. Tacrine was used as a positive control. Protein concentrations were measured with a kit from Bio-Rad (Hercules, CA, USA).

### 4.5. Synergism Calculation by the Median-Effect Principle

The interaction of herb/alkaloid synergy was evaluated by the median-effect principle [18,55]. The median effect equation (*F_a_*/*F_u_* = (*D*/*D_m_*)*^m^*) was used to calculate single or combined dose of herbs/alkaloids required for specific effect according to dose effective curves of single dose and their combination, in which *D* is the dose of drug, *F_a_* is the dose effect (equals to AChE activity inhibition showing with decimal fraction), *F_u_* is the unaffected fraction (*F_u_* = 1 − *F_a_*), *D_m_* is the dose required for 50% effect, and m is the slope of the linear equation (equal to the coefficient of the median effect equation). For convenience of computation, the median effect equation was taken as the logarithm of both sides and transformed to a linear equation: log(*F_a_*/*F_u_*) = *m*log*D* − *m*log*D_m_*. In addition, it was assumed that: *y* = log(*F_a_*/*F_u_*), *x* = log*D*, *a* = −*m*log*D_m_*; then, the linear equation (log(*F_a_*/*F_u_*) = *m*log*D* − *m*log*D_m_*) could be rewritten as a more convenient linear equation: *y* = *mx + a*. The linearity curves and equations of herbs/alkaloids were constructed by plotting the dose effect versus the log concentration of each dose effect. Hence, the *D_m_* of herbs/alkaloids was calculated according to the equation (*D_m_* = (*−a/m*)^10^). The combination index (CI) was used to evaluate the synergistic effect of pairing according to the Chou–Talalay equation (CI = (*D*)_1_/*(D_x_)*_1_ *+* (*D_x_*)_2_/(*D_x_*)_2_), in which, *D*_1_ and *D*_2_ are the dose of single herb/alkaloid required to produce the x% AChE active inhibition in their combination, respectively, while (*D_x_*)_1_ and (*D_x_*)_2_ was the dose of single herb/alkaloid in combination required to produce the same effect alone. (*D_x_*)_1_ and (*D_x_*)_2_ were similarly calculated according to the effect *F_a_* (*y* = log(*F_a_*/*F_u_*)) by linear equation (*y* = *mx + a*), after which, the CI values of herbs/alkaloids pair at different doses effect could be calculated. The values of CI < 1, CI = 1, and CI > 1 refer to a synergistic, additive, and antagonistic effect, respectively.

### 4.6. Molecular Docking

To investigate the underlying binding mechanisms of fangchinoline, coptisine, and berberine with AChE protein, we performed the molecular docking simulation using the MOE Dock module. The AChE 3D structure [19] was obtained from the Protein Data Bank (PDB code: 1eve), and its structure was prepared by correcting the structure issues (such as break bonds, missing loops, etc.), adding hydrogens, and calculating partial charges. The binding pocket was established using the binding site of cocrystalized AChE ligand 1-benzyl-4-[(5,6-dimethoxy-1-indanon-2-yl) methyl]-piperidine. The structures of fangchinoline, coptisine, and berberine were built with MOE Builder module and converted to 3D structures through energy minimization. Classical triangle matching was selected for the placement method, the output docking poses were evaluated by the default London dG score. Then, the rigid receptor method, keeping the ligand-binding groove rigid, was employed in the refinement step. The number of placement and refinement poses was set to 200 and 50, respectively, then the poses were submitted to minimizing using Amber10: EHT forcefield in MOE. The binding mode and the ligand–protein interaction of fangchinoline, coptisine, and berberine were analyzed in MOE after the refinement minimization.

### 4.7. Statistical Analysis

Statistical tests were done by DPS 18.10 software, using the bioassay module [56]. Data was expressed as the Mean ± SEM of three independent experiments, with triplicates of each experiment. The multiple drug effect analysis was used to evaluate the drug interaction according to the median-effect principle, as described in Section 2.5.

## 5. Conclusions

Both STR–CR and STR–PCC paired herbs showed synergistic effects on AChE inhibition, and the main alkaloids in the herbal extracts showed good synergistic effects. Fangchinoline–coptisine combinations (at ratios of 1:5, 1:2, and 1:1) and fangchinoline–berberine combinations (at ratios of 1:5, 1:2, and 2:1) showed good synergistic effects on AChE inhibition. Among the different ratios of fangchinoline–coptisine/berberine combinations, the ratio of 1:5 showed a better synergistic effect on AChE inhibition than the other ratios. In addition, the molecular docking results showed that fangchinoline, coptisine, and berberine could bind with the AChE enzyme in the active pocket site, which might result in the synergistic effect on AChE inhibition.

## Figures and Tables

**Table 1:** The linearity curves of the main alkaloids in STR, CR, and PCC.

Alkaloids	Linear Equation	*R* ^2^	Linear Range (μg/mL)
Fangchinoline	y = 7541.7x − 5907.9	0.9996	3.125–200
Tetrandrine	y = 6713x − 21313	0.9999	6.25–400
Coptisine	y = 37028x − 14419	0.9999	1.171875–75
Berberine	y = 46365x − 54509	0.9998	4.6875–300
Palmatine	y = 40443x − 19298	0.9998	1.171875–75
Epiberberine	y = 32555x − 8332.1	0.9998	0.625–40

The linearity curves were constructed by plotting the peak area versus the concentration of each analyte. Each regression equation was derived from six data points (*n* = 6).

**Table 2:** Acetylcholinesterase (AChE) inhibition of alkaloids in STR and CR.

Name	IC_50_	AChE Inhibitory Activity ^1^
STR extract	570.11 ± 10.41 μg/mL	+
CR extract	4.11 ± 0.15 μg/mL	+++
PCC extract	22.19 ± 3.12 μg/mL	++
Fangchinoline	2.17 ± 0.05 μM	+++
Tetrandrine	−	−
Coptisine	13.50 ± 1.48 μM	+
Berberine	2.33 ± 0.16 μM	+++
Palmatine	6.52 ± 0.84 μM	++
Epiberberine	18.70 ± 0.83 μM	+
Tacrine ^2^	0.44 ± 0.12 μM	+++

AChE inhibitory activity ^1^: “+” to “+++” indicate the ranking of AChE inhibitory activity; for molar concentration, “+++”, “++”, and “+” represent IC_50_ values of 1–5, 5–10 and 10–20 μM, respectively; for mass concentration, “+++”, “++”, and “+” represent IC_50_ values of 1–10, 10–100 and 100–600 μg/mL respectively; “−” indicates no effect. Tacrine ^2^: TAC served as a positive control. Data was expressed as Mean ± SEM, *n* = 3, each with triplicate samples.

**Table 3:** The binding residues of fangchinoline, coptisine, and berberine with the AChE active pocket.

Name	Alkaloids’ Binding Interactions with AChE				Scores ^1^
	Ligand	Receptor	Interaction	Distance Å	
Fangchinoline	O 4	O TYR_33__4_ (A)	H-donor	2.59	−7.11 ± 0.32
	6-ring	N PHE_288_ (A)	π–H	3.56	
Coptisine	C 9	6-ring PHE_330_ (A)	H–π	3.99	−6.76 ± 0.02
	C 30	6-ring TRP_279_ (A)	H–π	3.90	
Berberine	C 9	6-ring PHE_330_ (A)	H–π	4.03	−7.00 ± 0.01
	C 11	6-ring TYR_334_ (A)	H–π	3.57	
	C 13	6-ring TRP_279_ (A)	H–π	3.67	
Donepezil ^2^	C 2	6-ring TYR_334_ (A)	H–π	4.13	−7.99 ± 0.02
	C 18	6-ring TRP_279_ (A)	H–π	3.45	
	C 20	5-ring TRP_279_ (A)	H–π	4.03	
	C 28	6-ring TRP_84_ (A)	H–π	4.06	
	6-ring	6-ring PHE_330_ (A)	π–π	3.54	

^1^ The fitting score is expressed as Mean ± SEM, *n* = 3, and was negatively correlated with fitting effect. ^2^ Donepezil served as the positive control in docking.

## References

[1] Tsim K.W., Soreq H. (2012). Acetylcholinesterase: Old questions and new developments. Front. Mol. Neurosci..

[2] Campanari M.L., García-Ayllón M.S., Blazquez-Llorca L., Luk W.K., Tsim K.W., Sáez-Valero J. (2014). Acetylcholinesterase protein level is preserved in the Alzheimer’s brain. J. Mol. Neurosci..

[3] Dierckx R.I., Vandewoude M.F. (2008). Donepezil-related toxic hepatitis. Acta Clin. Belg..

[4] Castellino S.M., Tooze J.A., Flowers L., Hill D.F., McMullen K.P., Shaw E.G., Parsons S.K. (2012). Toxicity and efficacy of the acetylcholinesterase (AChE) inhibitor donepezil in childhood brain tumor survivors: A pilot study. Pediatr. Blood Cancer.

[5] Yang Z.D., Zhang D.B., Ren J., Yang M.J., Li S. (2012). Acetylcholinesterase inhibitory activity of the total alkaloid from traditional Chinese herbal medicine for treating Alzheimer’s disease. Med. Chem. Res..

[6] Murray A.P., Faraoni M.B., Castro M.J., Alza N.P., Cavallaro V. (2013). Natural AChE Inhibitors from Plants and their Contribution to Alzheimer’s Disease Therapy. Curr. Neuropharmacol..

[7] Kaufmann D., Kaur Dogra A., Tahrani A., Herrmann F., Wink M. (2016). Extracts from traditional Chinese medicinal plants inhibit acetylcholinesterase, a known Alzheimer’s disease target. Molecules.

[8] Gao Q., Li J., Cheung J.K., Duan J., Ding A., Cheung A.W., Zhao K., Li W.Z., Dong T.T., Tsim K.W. (2007). Verification of the formulation and efficacy of Danggui Buxue Tang (a decoction of Radix Astragali and Radix Angelicae Sinensis): An exemplifying systematic approach to revealing the complexity of Chinese herbal medicine formulae. Chin. Med..

[9] Gao Q.T., Cheung J.K., Li J., Chu G.K., Duan R., Cheung A.W., Zhao K.J., Dong T.T., Tsim K.W. (2006). A Chinese herbal decoction, Danggui Buxue Tang, prepared from Radix Astragali and Radix Angelicae Sinensis stimulates the immune responses. Planta Med..

[10] Choi R.C., Gao Q.T., Cheung A.W., Zhu J.T., Lau F.T., Li J., Li W.Z., Chu G.K., Duan R., Cheung J.K. (2011). A chinese herbal decoction, danggui buxue tang, stimulates proliferation, differentiation and gene expression of cultured osteosarcoma cells: Genomic approach to reveal specific gene activation. Evid. Based Complement. Altern. Med..

[11] Zheng K.Y., Choi R.C., Guo A.J., Bi C.W., Zhu K.Y., Du C.Y., Zhang Z.X., Lau D.T., Dong T.T., Tsim K.W. (2012). The membrane permeability of Astragali Radix-derived formononetin and calycosin is increased by Angelicae Sinensis Radix in Caco-2 cells: A synergistic action of an ancient herbal decoction Danggui Buxue Tang. J. Pharm. Biomed. Anal..

[12] Song Y.M., Li Z. (2006). Study of Fangji-Huangbai gel on analgesic and anti-inflammatory effects. J. Liaoning Univ. Tradit. Chin. Med..

[13] Liu Y., Deng A.J., Li X.F., Li Z.H., Zhang J.L., Du G.H., Qin H.L. (2009). Exclusive control substance of radix Stephaniae tetrandrae. Chin. J. Chin. Mater. Med..

[14] Chen M.L., Xian Y.F., Ip S.P., Tsai S.H., Yang J.Y., Che C.T. (2010). Chemical and biological differentiation of Cortex Phellodendri Chinensis and Cortex Phellodendri Amurensis. Planta Med..

[15] Choi H.S., Kim H.S., Min K.R., Kim Y., Lim H.K., Chang Y.K., Chung M.W. (2000). Anti-inflammatory effects of fangchinoline and tetrandrine. J. Ethnopharmacol..

[16] Sun Y.F., Wink M. (2014). Tetrandrine and fangchinoline, bisbenzylisoquinoline alkaloids from Stephania tetrandra can reverse multidrug resistance by inhibiting P-glycoprotein activity in multidrug resistant human cancer cells. Phytomedicine.

[17] Jung H.A., Min B.S., Yokozawa T., Lee J.H., Kim Y.S., Choi J.S. (2009). Anti-Alzheimer and antioxidant activities of Coptidis Rhizoma alkaloids. Biol. Pharm. Bull..

[18] Mak S., Luk W.W., Cui W., Hu S.Q., Tsim K.W., Han Y.F. (2014). Synergistic inhibition on acetylcholinesterase by the combination of berberine and palmatine originally isolated from Chinese medicinal herbs. J. Mol. Neurosci..

[19] Kryger G., Silman I., Sussman J.L. (1999). Structure of acetylcholinesterase complexed with E2020 (Aricept): Implications for the design of new anti-Alzheimer drugs. Structure.

[20] Checler F., Turner A.J. (2012). Special issue on Alzheimer’s disease: ‘amyloid cascade hypothesis-20 years on’. J. Neurochem..

[21] Schliebs R., Arendt T. (2006). The significance of the cholinergic system in the brain during aging and in Alzheimer’s disease. J. Neural Transm..

[22] Xie Q., Tang Y., Li W., Wang X., Qiu Z. (2006). Investigation of the binding mode of (−)-meptazinol and bis-meptazinol derivatives on acetylcholinesterase using a molecular docking method. J. Mol. Model..

[23] Wang X.P., Ding H.L. (2008). Alzheimer’s disease: Epidemiology, genetics, and beyond. Neurosci. Bull..

[24] Churcher I. (2006). Tau therapeutic strategies for the treatment of Alzheimer’s disease. Curr. Top. Med. Chem..

[25] Morgan C., Colombres M., Nuñez M.T., Inestrosa N.C. (2004). Structure and function of amyloid in Alzheimer’s disease. Prog. Neurobiol..

[26] Giacobini E. (2003). Cholinesterases: New roles in brain function and in Alzheimer’s disease. Neurochem. Res..

[27] Kar S., Slowikowski S.P., Westaway D., Mount H.T. (2004). Interactions between beta-amyloid and central cholinergic neurons: Implications for Alzheimer’s disease. J. Psychiatry Neurosci..

[28] Das A., Dikshit M., Nath C. (2005). Role of molecular isoforms of acetylcholinesterase in learning and memory functions. Pharmacol. Biochem. Behav..

[29] Descarries L., Aznavour N., Hamel E. (2005). The acetylcholine innervation of cerebral cortex: New data on its normal development and its fate in the hAPP (SW, IND) mouse model of Alzheimer’s disease. J. Neural Transm..

[30] Martini F., Pesarico A.P., Brüning C.A., Zeni G., Nogueira C.W. (2018). Ebselen inhibits the activity of acetylcholinesterase globular isoform G4 in vitro and attenuates scopolamine-induced amnesia in mice. J. Cell. Biochem..

[31] Inestrosa N.C., Alvarez A., Pérez C.A., Moreno R.D., Vicente M., Linker C., Casanueva O.I., Soto C., Garrido J. (1996). Acetylcholinesterase accelerates assembly of amyloid-beta-peptides into Alzheimer’s fibrils: Possible role of the peripheral site of the enzyme. Neuron.

[32] Rees T., Hammond P.I., Soreq H., Younkin S., Brimijoin S. (2003). Acetylcholinesterase promotes beta-amyloid plaques in cerebral cortex. Neurobiol. Aging.

[33] Alvarez A., Bronfman F., Pérez C.A., Vicente M., Garrido J., Inestrosa N.C. (1995). Acetylcholinesterase, a senile plaque component, affects the fibrillogenesis of amyloid-beta-peptides. Neurosci. Lett..

[34] Alvarez A., Opazo C., Alarcón R., Garrido J., Inestrosa N.C. (1997). Acetylcholinesterase promotes the aggregation of amyloid-beta-peptide fragments by forming a complex with the growing fibrils. J. Mol. Biol..

[35] Pavlov V.A., Parrish W.R., Rosas-Ballina M., Ochani M., Puerta M., Ochani K., Chavan S., Al-Abed Y., Tracey K.J. (2009). Brain acetylcholinesterase activity controls systemic cytokine levels through the cholinergic anti-inflammatory pathway. Brain Behav. Immun..

[36] Shaked I., Meerson A., Wolf Y., Avni R., Greenberg D., Gilboa-Geffen A., Soreq H. (2009). MicroRNA-132 potentiates cholinergic anti-inflammatory signaling by targeting acetylcholinesterase. Immunity.

[37] Durairajan S.S., Huang Y.Y., Yuen P.Y., Chen L.L., Kwok K.Y., Liu L.F., Song J.X., Han Q.B., Xue L., Chung S.K. (2014). Effects of Huanglian-Jie-Du-Tang and its modified formula on the modulation of amyloid-β precursor protein processing in Alzheimer’s disease models. PLoS ONE.

[38] Sangha J.S., Sun X., Wally O.S., Zhang K., Ji X., Wang Z., Wang Y., Zidichouski J., Prithiviraj B., Zhang J. (2012). Liuwei Dihuang (LWDH), a traditional Chinese medicinal formula, protects against β-amyloid toxicity in transgenic Caenorhabditis elegans. PLoS ONE.

[39] Sun Z.K., Yang H.Q., Chen S.D. (2013). Traditional Chinese medicine: A promising candidate for the treatment of Alzheimer’s disease. Transl. Neurodegener..

[40] Cai Z., Wang C., Yang W. (2016). Role of berberine in Alzheimer’s disease. Neuropsychiatr. Dis. Treat..

[41] Naaz H., Singh S., Pandey V.P., Singh P., Dwivedi U.N. (2013). Anti-cholinergic alkaloids as potential therapeutic agents for Alzheimer’s disease: An in silico approach. Indian J. Biochem. Biophys..

[42] Ng Y.P., Or T.C., Ip N.Y. (2015). Plant alkaloids as drug leads for Alzheimer’s disease. Neurochem. Int..

[43] He L.N., Liu Y.W., Yan Z.G., Huang S.P. (2011). Clinical study on improving cognition of patients with vascular dementia by Fangji Dihuang Decoction. J. Sichuan Tradit. Chin. Med..

[44] Liu N. (2016). Observe on the curative effect of Huanglian Jiedu decoction combined with Tianwang Buxin Dan in the treatment of heart and liver Yin deficiency type senile dementia. Mod. J. Integr. Tradit. Chin. West. Med..

[45] Xiong X.J. (2019). Fangji Dihuang Decoction formula syndrome and Fengyin decoction formula syndrome: Application in stroke and mental disorders. China J. Chin. Mater. Med..

[46] Khan T., Ahmad R., Azad I., Raza S., Joshi S., Khan A.R. (2018). Computer-aided drug design and virtual screening of targeted combinatorial libraries of mixed-ligand transition metal complexes of 2-butanone thiosemicarbazone. Comput. Biol. Chem..

[47] Cai Z., Wang C., He W., Chen Y. (2018). Berberine Alleviates amyloid-beta pathology in the brain of APP/PS1 transgenic mice via inhibiting β/γ-secretases activity and enhancing α-secretases. Curr. Alzheimer Res..

[48] Lu D.Y., Tang C.H., Chen Y.H., Wei I.H. (2010). Berberine suppresses neuroinflammatory responses through AMP-activated protein kinase activation in BV-2 microglia. J. Cell. Biochem..

[49] Lin T.Y., Lu C.W., Tien L.T., Chuang S.H., Wang Y.R., Chang W.H., Wang S.J. (2009). Fangchinoline inhibits glutamate release from rat cerebral cortex nerve terminals (synaptosomes). Neurochem. Int..

[50] Cho S.O., Seong Y.H. (2002). Protective effect of fangchinoline on cyanide-induced neurotoxicity in cultured rat cerebellar granule cells. Arch. Pharm. Res..

[51] Yu D., Tao B.B., Yang Y.Y., Du L.S., Yang S.S., He X.J., Zhu Y.W., Yan J.K., Yang Q. (2015). The IDO inhibitor coptisine ameliorates cognitive impairment in a mouse model of Alzheimer’s disease. J. Alzheimers Dis..

[52] Ji H.F., Shen L. (2011). Berberine: A potential multipotent natural product to combat Alzheimer’s disease. Molecules.

[53] Ellman G.L., Courtney K.D., Andres V.J., Feather-Stone R.M. (1961). A new and rapid colorimetric determination of acetylcholinesterase activity. Biochem. Pharmacol..

[54] Tsim K.W., Randall W.R., Barnard E.A. (1988). An asymmetric form of muscle acetylcholinesterase contains three subunit types and two enzymic activities in one molecule. Proc. Natl. Acad. Sci. USA.

[55] Chou T.C. (2010). Drug combination studies and their synergy quantification using the Chou-Talalay method. Cancer Res..

[56] Tang Q.Y., Zhang C.X. (2013). Data Processing System (DPS) software with experimental design, statistical analysis and data mining developed for use in entomological research. Insect Sci..

